# Patient attitudes, beliefs, and practices in environmentally friendly inhaler disposal: a mixed-methods evaluation with behaviourally informed recommendations

**DOI:** 10.1038/s41533-026-00521-6

**Published:** 2026-05-22

**Authors:** Delyth James, Terence J. McElvaney, Thomas Henstock, Hannah Thomas, Aleysha Caffoor, Alys Harrop, Sarah Brown, Catherine Heidi Seage

**Affiliations:** 1 School of Medicine, Swansea University, Singleton Campus, Swansea, SA2 8PL Wales UK; 2 Cardiff School of Sport & Health Sciences, Cardiff Metropolitan University, Llandaff Campus, Cardiff, Wales CF5 3YP UK; 3 Strategic Analysis, Data & Analytics, NHS England, Wellington House, 133-155 Waterloo Rd, London, England SE1 8UG UK; 4 Public Health Wales, Capital Quarter 2, Cardiff, Wales CF10 4BZ UK

**Keywords:** Environmental sciences, Health care

## Abstract

The National Health Service (NHS) aims to reduce emissions from inhalers, a major contributor to its carbon footprint. Despite willingness, patient awareness and engagement with appropriate inhaler disposal and recycling remains low. This paper combines two studies both using the Capability, Opportunity, Motivation-Behaviour (COM-B) model with contrasting methodological approaches to explore inhaler disposal behaviours, and barriers and facilitators to recycling uptake. Synthesis of two datasets from quantitative (Study 1) and qualitative (Study 2) studies was undertaken, using deductive analysis (i.e. categorisation of barriers/facilitators from both studies and comparison to each COM-B component) to identify patterns across both studies inhaler users were unaware of the need to return inhalers to pharmacies for disposal and often discarded them in domestic waste. Inhaler users viewed community pharmacies as convenient locations for recycling schemes due to their proximity to the local pharmacy. However, they were unlikely to engage with initiatives that require significant additional effort such as traveling out of their way to return inhalers. Although awareness of environmental impacts can motivate intentions to recycle, users reported struggling to develop and maintain consistent recycling habits. Barriers to appropriate inhaler disposal were therefore evident across psychological capability (limited knowledge), physical opportunity (access and convenience), and automatic motivation (habitual disposal practices). These findings highlight persistent gaps between positive environmental intentions and actual disposal behaviours among inhaler users and the complexity of behaviour change, requiring interventions that address psychological, physical, motivational, environmental and social barriers. Future research should focus on implementing behaviourally informed approaches and evaluating these interventions to identify the most effective methods for improving inhaler disposal and reducing the environmental impact of inhaler use.

## Introduction

By 2026, the National Health Service (NHS) is expected to deliver a 50% reduction in carbon emissions produced from waste management^1^ rising to 80% by 2028–2032^1,2^. Metered dose inhalers (MDIs) are one of the greatest single contributors to carbon emissions across the NHS^3,4^ since they contain hydrofluorocarbon (HFCs) propellants, estimated to account for 4% of all NHS carbon dioxide (CO*2)* emissions. This compares to 14% of emissions attributed to the entire NHS transport fleet and 3% associated with food and catering^5^. One estimate is that 73 million inhalers are dispensed every year^6^. There is a lack of data regarding the current disposal behaviours of inhaler users. Various initiatives have been proposed to reduce the significant global warming impact from inappropriate MDI disposal^7^ involving prescribing less inhalers^8^, switching to a non-HFC inhaler^3,9^ or the implementation of recycling schemes^7,10^. Inhaler recycling involves collection and disposal of MDIs by a specialist waste contractor where the plastic, metal and gas are recovered and reused. This inhaler recycling technology has already been successfully used in a small number of trials run by NHS organisations across England and Wales and in projects run by two major pharmaceutical companies^11^. One example is a project that took place in community pharmacies and hospitals across Leicestershire, England, estimated to have saved the equivalent of 305.3 tonnes of CO_2_ emissions from entering the atmosphere where 52,148 inhalers were received via postal return over a 24-month period^7^.

To our knowledge there are currently no centralised national schemes in place in the United Kingdom (UK) utilising this or alternative approaches to dispose of these inhalers in an environmentally friendly way. Patients are encouraged to return all unused and empty inhaler canisters (along with all other unwanted medicines) to the community pharmacy for safe disposal. Currently, these are incinerated along with all returned medicinal waste; however, the temperatures are not high enough to prevent carbon emissions from the MDIs, even if the canisters are empty, therefore still contributing to carbon emissions. The future implementation of a community pharmacy inhaler recycling scheme relies on engaging with patients to encourage environmentally friendly inhaler disposal. However, these changes will also require investment in system-level infrastructure to link community pharmacies with specialist waste contractors to effectively reduce CO_2_ emissions.

Several studies highlight limited public awareness regarding the environmental impact of inhalers. Hodge et al.^12^ found that 71% of inhaler users (*n* = 61) were unaware that inhalers contain greenhouse gases. Similarly, over 80% of patients recruited from UK hospitals and GP surgeries were unaware that inhalers could be recycled^13^. While a high proportion of users (93%) expressed a desire for more environmental information about MDIs^14^, fewer than 15% of healthcare professionals routinely discussed inhaler recycling with their patients^15^.

Other studies have shown that despite low initial awareness, many patients are open to more sustainable practices, expressing a willingness to recycle if accessible facilities are made available^16^. Murphy et al’s^7^ pilot postal return scheme showed high patient satisfaction, but initial uptake was slow, gradually increasing over time. Broader engagement with recycling initiatives remains limited. In a secondary care clinic, only 35% of patients (*n* = 261) recycled their inhalers over six months, while 57% continued to dispose of them in the household waste^17^. In paediatric and community settings, educational efforts improved awareness of the need to recycle inhalers, however, consistent engagement with recycling schemes remained a challenge^18^. International studies echo these trends, with improper disposal practices remaining prevalent despite media advertisements or financial incentives being used to influence behaviour^19,20^. Further to this, Murphy et al^21^ reported that over half of patients (*n* = 16/29; 55%) using an inhaler without a dose counter did not have the confidence to determine when their inhaler was empty, resulting in inhalers being returned either under or over-used.

Across the existing literature, there is consensus that patient and staff education, availability of recycling points, and clearer communication from healthcare providers and pharmaceutical companies are essential^5,16–18,21,22^. The NHS recommends incorporating recycling advice during consultations and harnessing general practice settings to promote inhaler disposal practices, to reduce the environmental impact and support sustainability goals^23^. Although system-level options for inhaler recycling are currently limited, before effective solutions can be proposed, we need to understand the current behaviours, beliefs and attitudes of inhaler users towards inhaler disposal and recycling. For this paper, the terms ‘appropriate inhaler recycling,’ ‘appropriate inhaler disposal’ and ‘environmentally friendly disposal’ refer to the behaviour of taking the used (or unused) inhaler back to the community pharmacy or engaging in an alternative inhaler recycling scheme such as the postal initiative described earlier.

Current guidance is that all unused or unwanted medicines should be returned to the pharmacy for appropriate disposal. Watkins et al^24^. showed poor public awareness that returning medicines to pharmacies is the correct disposal route, with many considering sink, toilet, or bin disposal acceptable—highlighting environmental harms beyond climate change (e.g. water contamination). Only MDIs require a specific process of incineration due to their significant carbon emissions, yet this approach to their disposal is not currently routine practice across pharmacies in the UK. Existing literature suggests that general attitudes towards inhaler waste should not be conflated with knowledge and engagement relating to inhaler recycling schemes^10,25^. Evidence suggests that low awareness of pharmacy return routes for medicines in general^26^ underpins much inappropriate inhaler disposal, while participation in inhaler recycling schemes requires additional, scheme‑specific knowledge, motivation, and opportunity. This reinforces the need to take a behaviourally informed approach to understanding patient inhaler disposal practices.

### The Behaviour Change Wheel

**Fig. 1:**
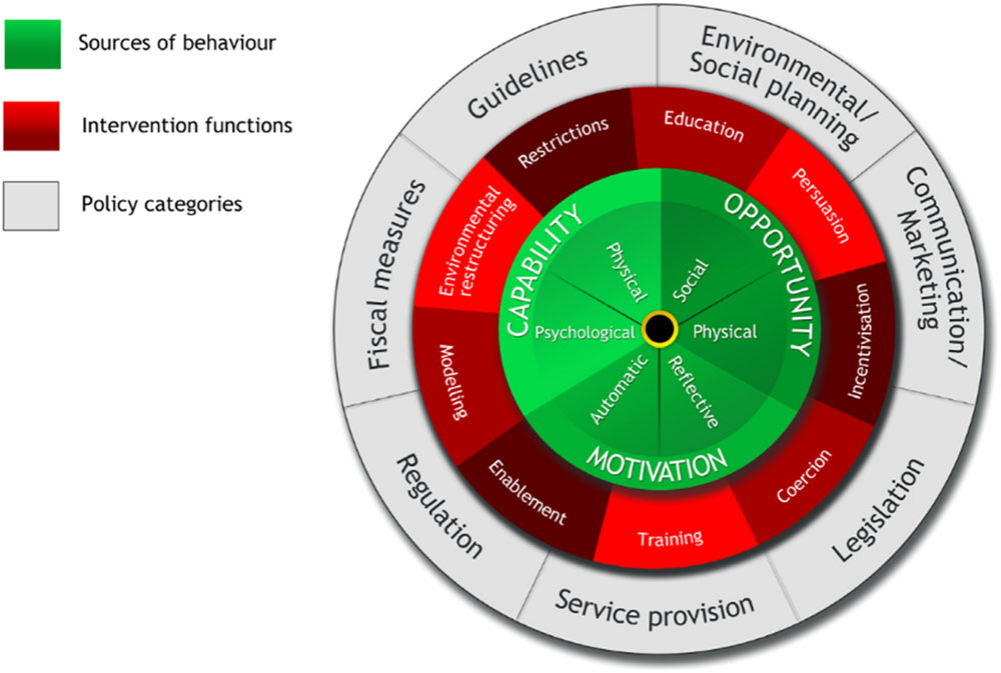
The Behaviour Change Wheel (BCW)^27^.

The BCW was developed to improve the design and implementation of behaviour change interventions^27^ by providing a method of characterising interventions and linking them to target behaviours. The COM-B model components lie at the core of the BCW, enabling barriers and facilitators to be mapped to appropriate intervention functions. The policy categories on the outer circle of the BCW are key to influencing change and are essential considerations for successful interventions (Fig. 1).

**Fig. 2:**
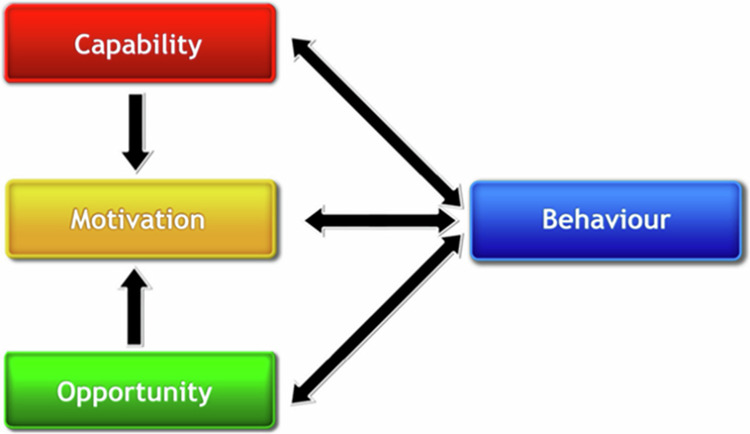
*The COM-B model – a framework for understanding behaviour*^27^.

The COM-B model allows the barriers to specific behaviours (i.e. environmentally friendly inhaler disposal) to be systematically explored (Fig. 2). The COM-B model is based on the concept that the interaction between several components, namely, the individual’s capability (C), opportunity (O) and motivation (M) can provide an explanation for why a particular behaviour (B) is or is not performed^28^. For an intervention to be successful, one or more of these components need to be targeted for sustained behaviour change to occur.

For the purpose of this paper, the target population is patients (or guardians) prescribed an MDI and the target behaviour is consistently taking the inhaler (canister plus plastic case) back to a community pharmacy regardless of how it is disposed. We present and integrate the analysis of two complementary studies, both underpinned by behavioural science theory, conducted independently of each other, adopting two contrasting methodological approaches to assess the current rates of appropriate inhaler disposal, and attitudes and beliefs about inhaler recycling. The synthesis sought to identify converging and diverging findings from both studies and use multiple data sources to help build a more complete understanding of inhaler recycling behaviours. This entailed mapping the findings across both quantitative and qualitative data to the COM-B model to gain a comprehensive understanding of the barriers and facilitators to community pharmacy inhaler recycling that neither study could reveal in isolation. This also allowed us to compare the findings from two contrasting methodological approaches to identify areas of divergence or similarities. Importantly the synthesis of findings allowed us to identify key behavioural targets which were common to both studies and take this forward into the design of future interventions to improve patient inhaler recycling practices.

The first study (unpublished) was a quantitative evaluation of people’s awareness of the environmental impact of inhalers, their current inhaler disposal behaviours, and the factors impacting appropriate current and future inhaler disposal. The second study^10^ (mapping to COM-B – published; thematic analysis presented in this paper - previously unpublished) was a qualitative evaluation of patients’ perspectives on returning used or unused inhalers to a community pharmacy with the aim of exploring the perceived barriers and facilitators to appropriate inhaler disposal (i.e. returning to a pharmacy or engaging with another recycling scheme). Both studies, therefore, investigated the same behaviours in a similar population, within a comparable timeframe in an area embedded in NHS policy. Both studies were underpinned by two inter-related psychological frameworks – the BCW and COM-B model of behaviour^27^. COM-B was used to guide the design of questions and diagnose the behavioural drivers of inhaler disposal practices, while the BCW guided the design of a behaviourally informed message that was experimentally tested within Study 1. The integration and synthesis of both datasets was also structured around the COM-B model and BCW^27^ using framework analysis^29^. This followed the principles and practices for achieving integration in mixed methods design by merging of data, where one study was not informed by the other^30^.

### Aims and objectives

The aim of this paper is to present and synthesise the findings from two independent studies to assess current inhaler users’ attitudes, beliefs, and practices related to inhaler disposal. To achieve this aim, the following objectives were set:

1. To categorise barriers and facilitators identified from two studies, compare the findings at each framework (i.e. COM-B model) level and identify patterns across both studies.
2. To map the synthesis of findings from both studies to the BCW to make behaviourally informed recommendations to support appropriate inhaler disposal behaviours.

## Methods

### Study design and participant recruitment

Study 1: Data were collected using an online quantitative survey (hosted via Qualtrics™ between 09/04/2024 and 28/04/2014) developed to assess inhaler disposal practices, willingness to return inhalers to the pharmacy and the perceived barriers and facilitators to inhaler disposal. (See Supplementary Material 2 for details on questionnaire design). Participants were recruited using a volunteer sampling approach. The survey link was distributed via a study advertisement email sent to members of the NHS App and NHS Vaccine participant panels—national panels comprising individuals who have registered their willingness to be contacted by the NHS for research and service‑evaluation activities.

Study 2: The study adopted qualitative methodology and was advertised via posters in community pharmacies and social media adverts (X™’, Facebook™). Participants were given a £20 gift voucher to show appreciation for their time. A sample of 11 participants was appropriate for this study, given that framework analysis emphasises analytic rigour, case‑based comparison, and theoretical adequacy over sample size or data saturation, and is commonly applied to small, purposively selected samples in health services research^31,32^.

For both studies, eligible participants were inhaler users (or their guardian) aged 18 years or older, prescribed one or more MDI inhalers. Convenience sampling was adopted for both studies where participants needed to be living in England for inclusion in Study 1 or one of three areas of Wales (North, Southeast and Southwest) for inclusion in Study 2.

### Ethics

Study 1: This study met the Health Research Authority (HRA) criteria for service evaluation^33^ as its sole purpose was to assess and improve the quality of the existing NHS inhaler communication and future development of disposal services. It did not alter clinical care or test a clinical intervention but aimed to deliver improvements in NHS understanding of patient engagement with the service. Consequently, NHS HRA Approval/ Research Ethics Committee reviews were not required. Procedures were put in place to ensure lawful and ethical handling of participant data adhering to General Data Protection Regulation (GDPR) requirements^34^.

Study 2: Cardiff Metropolitan University, Cardiff School of Sport & Health Sciences, Ethics Committee (Reference number: Sta-7736) granted ethical approval, with additional approval granted to combine the datasets of both studies for the purpose of synthesis and further analysis (Reference number: sta-10739).

### Data collection

**Fig. 3:**
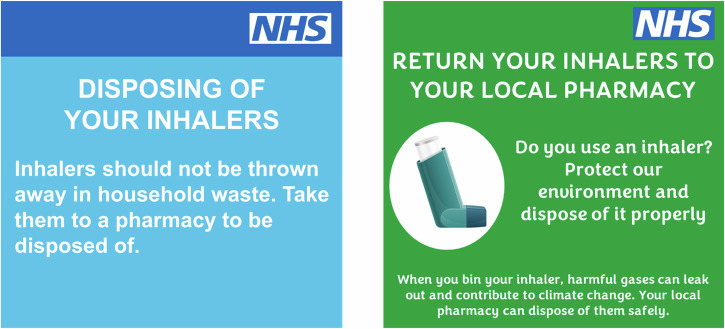
Message A (left), based on existing NHS communications and Message B (right).

Study 1: A multi-stage quantitative survey (Supplementary Material 1) was designed to gather current inhaler disposal practices and views about inhaler recycling. The opportunity for free-text responses was also provided for some questions. An information sheet and eligibility screening questions were included and informed consent was captured. Eligible participants indicated from a list of options how they currently dispose of their inhalers and reasons for doing so (free text). A multiple-choice question (MCQ) assessed their understanding of NHS inhaler disposal recommendations. Following a between-participant design, half of the respondents were presented with a standard NHS message about inhaler disposal (Message A) and half a behaviourally informed message about inhaler disposal (Message B) (Fig. 3). This message test aimed to ascertain whether the framing of inhaler disposal messaging impacts future likelihood of proper disposal by comparing a behaviourally informed message to current NHS communications (See Supplementary Material 2). Participants rated their likelihood of returning inhalers in the future on a scale of 1–7. Those scoring 4 or below were presented with specified options to select reasons for not returning inhalers in the future. Awareness of inhalers’ environmental impact was assessed by asking participants to estimate how many miles they would need to drive in an average petrol car to have the same carbon footprint of a presented inhaler. Participants made these estimates for both a pressurized MDI and DPI, where the order of presentation of questions was randomized. Questions regarding the types of inhalers used, guidance received on disposal, perceived importance of reducing inhaler carbon footprint, and support needed for proper future disposal were included. Basic demographic information was also collected.

Study 2: One-to-one semi-structured interviews were conducted (by AC and AH) online (via MS Teams™) in Autumn 2023. (Both researchers were female health psychology postgraduate students with experience of conducting qualitative research related to asthma. AH also had experience of reviewing asthma medication in a GP setting and had asthma as a child. The interviewer and interviewees had no prior relationships. All those who consented to taking part completed the interviews. An interview schedule (Supplementary Material 3) was developed based on existing literature, the COM-B model of behaviour^27^ and Theoretical Domains Framework (TDF)^35,36^ as a guiding framework. Introductory questions were used to gain an understanding of inhaler users’ experiences of inhaler recycling and their knowledge of the environmental impact of inappropriate disposal of inhalers. The interviews explored perceptions of the barriers and facilitators to appropriate inhaler disposal and recycling in a community pharmacy. Interviews were audio-recorded, anonymised and transcribed verbatim for analysis^10^ and lasted between 18–60 (mean 35) minutes. Transcripts were not returned to participants for review or correction. (See COREQ checklist for reporting qualitative methods in Supplementary Material 4).

### Data analysis

Study 1: All statistical analyses were carried out using RStudio Version 12.0. Descriptive statistics were generated for all recorded variables. One-sample Wilcoxon signed rank tests were used to compare reported estimated carbon footprint mileage equivalents of MDIs and DPIs to their actual mileage equivalents. A Mann-Whitney U test was used to compare likelihood of returning inhalers in the future in response to Message A vs Message B. Likelihood of returning inhalers was further modelled with a binary logistic regression using dichotomized responses, with scores from 1–4 vs 5–7 on the likelihood scale coded as 0 and 1 respectively. We focused on the maximal category as this was best placed to give an indication as to whether messaging could shift people from general agreement to unequivocal commitment, which is the level of intention most likely to translate into actual behaviour. Additionally, responses were heavily skewed toward the top of the scale, creating ceiling effects. Thus, dichotomising at the highest value provided a cleaner and more meaningful behavioural threshold. Robustness checks were conducted using ordinal logistic regressions, retaining the full 7-point scale. As these checks did not impact the findings, results are reported from the binary logistic regressions.

Study 2: Initially inductive thematic analysis of all barriers and enablers was undertaken^37^. As reported in Seage et al^10^, deductive framework analysis^29,38^ was undertaken by mapping the interview data to the COM-B model followed by thematic analysis of any data that did not map (all data were mapped to COM-B components in this case so further thematic analysis was not needed). Codes were applied to the framework’s pre-existing categorical themes (e.g., physical capability, psychological capability, etc.)^39^.

Synthesis of the quantitative (Study 1) and qualitative (Study 2) datasets was undertaken manually using deductive analysis (i.e. categorisation of barriers/facilitators from both studies and comparison of each COM-B component) to identify patterns across both studies. This is in line with recommendations for conducting inductive / deductive hybrid thematic analysis in mixed methods research^40^ and guidance on data reconfiguration (i.e. analysing both datasets in terms of COM-B) and transfer/exchange^41^. This was initially conducted by one researcher (HS) and independently verified (DJ). Both researchers have extensive experience in framework analysis and led the conceptualisation and operationalisation of Study 2. Any discrepancies were discussed with the Study 1 team (TMcE, TH) to ensure accurate interpretation of data and agreement to reach consensus.

## Results

Table 1 presents the relevant demographic and clinical characteristics of participants for both studies and their inhaler related practices.

**Table 1:** Comparison of key participant characteristics for Studies 1 and 2.

	Study 1 (Quantitative)	Study 2 (Qualitative)
**Number of participants**	*n* = 1354	*n* = 11
**Gender**	Male: 528 (39%) Female: 812 (60%) Other: 14 (1%)	Male: 7 (64%) Female: 4 (36%)
**Age**	Range = 18–95 years Mean = 60.9 years (*SD* = 13.85)	Range = 18–65 years Mean 41.2 years (SD = 11.75)
**Ethnicity**	White: 91% Black: 2.5% Asian: 2.5% Other: 4%	White: 9 (82%) Black: 2 (18%) Asian: 0 (0%) Other: 0 (0%)
**Inhaler user / Guardian**	Inhaler user = 1237 (91%) Guardian = 117 (9%)	Inhaler user = 10 (91%) Guardian = 1 (9%)
Diagnosis^*^	Asthma: 1029 (76%) COPD: 298 (22%) Other: 122 (9%)	Not ascertained
Inhaler types^*^	MDI = 1137 (84%) DPI = 433 (32%) SMI = 108 (8%)	MDIs = 11 (100%)
**Currently return inhalers to pharmacy**	284 (21%)	1 (9.1%)
**Awareness of:** Carbon footprint changes (Study 1)^**^ **Existence of recycling schemes (Study 2)** **Need for recycling (Study 2)**	199/1153 (17%) - -	- 0 (0%) 0 (0%)

^*^Some inhaler users had a diagnosis of both asthma and COPD or prescribed more than one type of inhaler which accounts totals over 100%. **Denominator lower due to attrition in survey respondent numbers.

*MDI* metered dose inhaler, *DPI* dry powder inhaler, *SMI* soft mist inhaler.

### Study 1: Quantitative Study

Most respondents were white, female, MDI users, an average age of 61 years with a diagnosis of asthma. Only a fifth currently returned their inhalers to the pharmacy and awareness of the carbon footprint implications was less than this (17%). Of the sample, the majority (79%; *n* = 1070) failed to return their inhalers to their local pharmacy with 44% (*n* = 596) reporting that they dispose of them in the general waste, and 29% (*n* = 393) disposing them in their standard recycling bin. When asked how they thought the NHS recommends they dispose of their inhalers, 70% (*n* = 948) of participants failed to correctly identify the pharmacy as the appropriate channel for disposal. When asked why they disposed of their inhalers in the way they currently did, over half (56%; *n* = 758) indicated that they simply did it out of habit, with less than a third (30%: *n* = 406) thinking that they were using the correct method. Nearly three-quarters of participants (74%; *n* = 1002) indicated that they had never received any guidance on how to dispose of their inhalers properly.

The desire to adopt pro-environmental behaviours was high, with the majority (84%; *n* = 1137) indicating that reducing their carbon footprint was either “Important” or “Very important” to them. When presented with messaging encouraging appropriate inhaler disposal, participants showed a high likelihood to return their inhalers to the pharmacy in future (Mean likelihood score = 6.24 out of 7, *SE* = 0.04). A Mann-Whitney U test found that participants who saw a behaviourally informed message, specifically designed for the study (Message B, Mean likelihood score = 6.31, *SE* = 0.05), were significantly (*U* = 19588.50, *p* = 0.008) more likely to return their inhalers than those who saw messaging based on existing NHS communications (Message A, mean = 6.17, *SE* = 0 0.05) (Fig. 3).

Those who indicated they were unlikely to return their inhalers (a likelihood of 4 or below on the 7-point likelihood scale) to the pharmacy in the future (10.7%; *n* = 143/1333) were also asked for their primary reasons. The most frequently reported reason (38%; *n* = 54/143) was related to the additional effort needed to return inhalers to the pharmacy. Other reasons cited as key barriers to appropriate disposal were forgetfulness (22%; *n* = 31/143) and recognising that changing their existing disposal habits might be challenging (13%; *n* = 19/143).

#### Predictors of inhaler disposal by returning to the pharmacy

Modelling with a binary logistic regression found several factors significantly predicted whether participants reported they would be likely (5 or above on a 7-point likelihood scale) to return their inhalers to the pharmacy in future (χ2(17) = 136.97, p < 0.001) (see Table S3 in Supplementary Material 5 for full regression results).. Those who saw Message B were significantly more likely to return their inhalers to the pharmacy in the future (*OR* = 1.61, 95% *CI* = [1.04, 2.50], *p* = 0.033). Participants who indicated that reducing their carbon footprint was “Very important” were more likely to return their inhalers to the pharmacy in the future (*OR* = 6.75, 95% *CI* = [4.06, 11.77], *p* < 0.001). Those who currently returned their inhalers to the pharmacy were more likely to continue to return them in future (*OR* = 28.07, 95% *CI* = [5.46, 516.84], *p* = 0.001).

#### Awareness of inhaler environmental impact

Participants were tasked with estimating the carbon footprint of an MDI relative to miles driven in a standard petrol car. A one-sample Wilcoxon signed rank test showed that participants significantly underestimated the carbon footprint, with a median response of 20 miles compared to the actual mileage equivalent of approximately 107 miles (*Z* = −16.35, *r* = −0.47, *p* < 0.001, *n* = 1211). Participants also significantly overestimated the relative carbon footprint of DPIs, with a median estimate of 10 miles compared to the actual equivalent of approximately 02 miles (*Z* = 25.96, *r* = 0.75, *p* < 0.001, *n* = 1206).

### Study 2: Qualitative study

All participants were white, MDI users (one guardian) ranging from 18–65 years old. Only one currently returned inhalers to the pharmacy and none was aware of the need to recycle. In this small group of 11 participants, three themes were identified across the entire dataset relating to barriers and enablers to safe disposal of inhaler medication by returning to a community pharmacy. Barriers were: Theme 1 – ‘*I’m not going out of my way’*; Theme 2 – ‘*No-one told us’*, and Theme 3 – ‘*Will it really make a difference?*’. Enablers were: Theme 1 - ‘*Recycling aligns with my values’*; Theme 2 –’*I already recycle, so it shouldn’t be a problem*’ and Theme 3 - ‘*How hard can it be?*’. Tables 2 and 3 provide illustrative quotes for each theme relating to barriers and enablers for inhaler recycling in the pharmacy. Examples were selected from the data to represent typical participant perspectives within each theme.

**Table 2:** Barriers to returning inhalers for recycling in the pharmacy: Illustrative quotes from qualitative interviews (*n* = 11).

Theme 1 - ‘*I’m not going out of my way’*	Theme 2 - ‘*No-one told us’*	Theme 3 - ‘*Will it really make a difference?*’
“*Anything extra, people are less likely to do because you are likely to forget*” (P2).	*“I’ve been taking pumps for 40 years now, longer than I’d care to admit, but it has never been pointed out to me that I could take the empty inhaler back to the pharmacy. And I’ve been through four or five pharmacies …just mention to people as they’re picking things up, you know…. you would argue it probably isn’t a huge thing to just say ‘you can drop these things back here, bring your old ones back and we’ll give you your new one’ or whatever.”* (P1).	*“Personally, I don’t think it’ll have much of an individual impact, if I recycle them or not, but I think with others it definitely will”* (P3).
*“You can’t take the old one in when you pick up the new one. So, you’d have to make, you’d almost have to make a special trip”* (P1).	*“I don’t think I’ve actually been told by a pharmacist that you can take anything back”* (P1).	*“So, the problem is that something like an inhaler, it seems so small and. You know, you look at the amount of recycling that you put out on a weekly basis and the amount of bin bags that people put out. Recycling, what you know, an inhaler once a month… I know it’s important, but you will struggle to persuade people that it’s important”* (P2).
*“So, when you’re tidying up, you think, oh, just chucking it in the bin, chucking it in the recycling. So yeah, that’s the problem.” (*P2).	*“I have asked and several others, you know, how do we dispose of inhalers? Have you got a scheme? And they sort of just look at me blank sort of well no just put them in the bin and that’s it”* (P2).	-
-	*“Before I saw your study, I had literally not thought about this…” (P7)*.	-
-	*“…in my pharmacy, personally, I don’t really see any posters or leaflets anywhere”* (P1).	-

**Table 3:** Enablers to returning inhalers for recycling in the pharmacy: Illustrative quotes from qualitative interviews (*n* = 11).

Theme 1 – ‘*Recycling aligns with my values’*	Theme 2 - ’*I already recycle, so it shouldn’t be a problem*’	Theme 3 - ‘*How hard can it be?’*
*I think it’s definitely important to take them where they need to go and recycle them properly”* (P1).	*“Yeah. cause we recycle everything else. We pride ourselves on the fact. And you know, we’re a family of four. And every fortnight we take out half a bin bag”* (P2).	“*I have to pick mine up from the pharmacy anyway. So, you know, it really doesn’t get much easier than that”* (P1).
*“I would be using it regardless of whether I get a reward or not; obviously it’s a nice benefit, but”…* (P3).	*“I like to make sure that I do recycle and stay up, stay up on sustainability”* (P4).	*. “I wouldn’t have thought so. I should imagine it would be simply in a matter of handing them over”* (P5).
*-*	*“I’m quite big on not using propellant as much as possible”* (P5).	*“Once a month we go there, so simply take anything that isn’t used and isn’t going to be used*. (P5).
-	“*I do generally try to dispose of all my waste correctly and recycle if possible. So shouldn’t really be any different for the inhaler”* (P3).	-

### Synthesis of findings from Studies 1 and 2

Tables 4–6 present the mapping of the barriers and facilitators from Studies 1 and 2 to the COM-B Model components, highlighting the commonalities across the data sets. Table 4 presents the findings relating to factors affecting participants’ capability of disposing of inhalers appropriately.

**Table 4:** Mapping of barriers and facilitators from Studies 1 and 2 relating to the Capability component of the COM-B Model.

Capability	Commonalities	Barriers identified in Study 1 survey data	Barriers identified in Study 2 interview data	Facilitators identified in Study 1 survey data	Facilitators identified in Study 2 Interview data
*Psychological Capability*	Knowledge and awareness of inhaler disposal and recycling	70% of participants did not know that the NHS recommends returning their inhaler to the pharmacy. Only 17% of respondents were aware that disposal method affected resultant emissions.	Participants were unaware that inhalers could/or should be recycled.	Participants who knew how they are supposed to dispose of inhalers were over 16 times more likely to currently return them to the pharmacy Participants who were aware that the method of inhaler disposal impacted emissions were over twice as likely to currently return them to the pharmacy. (See Table S1 in Supplementary Material 5 for full regression results).	None
*Physical Capability*	Physical demands for inhaler recycling to be undertaken	For the 11% of patients who said they would not return their inhalers in the future, 38% highlighted the additional effort required as a barrier. Free-text responses highlighted that some patients were not capable of making necessary visits to the pharmacy independently.	Participants perceived themselves to not have the skills needed to be able to recycle inhalers correctly at home. Additional effort needed to plan additional journeys to the pharmacy to return inhalers for recycling. Reliance on public transport to travel to get to the pharmacy.	None	Participants felt that they would have the necessary skills to return inhalers to the pharmacy.

**Table 5:** Mapping of barriers and facilitators from Studies 1 and 2 relating to the Opportunity component of the COM-B Model.

Opportunity	Commonalities	Barriers identified in Study 1 survey data	Barriers identified in Study 2 interview data	Facilitators identified in Study 1 survey data	Facilitators identified in Study 2 interview data
*Physical Opportunity*	Use of the pharmacy setting for recycling inhalers	Some free-text responses highlighted that the pharmacy is too far away or not open at convenient hours. Some pharmacies do not currently offer an inhaler disposal service.	Participants who use small, independent pharmacies perceived that their local pharmacy would not have the time, space, or resources to run a recycling scheme.	Older participants (over 60 yrs) were more likely to return inhalers, likely reflecting that they may have more regular visits to the pharmacy than younger inhaler users (See Table S1 in Supplementary Material 5 for full regression results). Free-text responses indicated that convenient disposal points would encourage them to recycle.	Inhaler users regularly visit the pharmacy and did not see handing inhalers back for recycling as onerous.
*Social Opportunity*	Healthcare professionals as a source of advice	74% said they’d never received any guidance, with 6% not recalling if they were given any guidance	Participants stated that healthcare professionals had not informed them about how to dispose of inhalers	Public messaging around this topic could shift social norms and expectations around proper disposal.	Pharmacists viewed as an appropriate professional role to encourage engagement with inhaler recycling,

**Table 6:** Mapping of barriers and facilitators from Studies 1 and 2 relating to the Motivation component of the COM-B Model.

Motivation	Commonalities	Barriers identified in Study 1 survey data	Barriers identified in Study 2 interview data	Facilitators identified in Study 1 survey data	Facilitators identified in Study 2 interview data
*Reflective Motivation*	Environmental impacts of engaging or not engaging in inhaler recycling.	Using median values, participants underestimated the carbon footprint of MDIs by 81% and overestimated the carbon footprint DPIs by 352%.	Some patients did not feel that their individual contribution to recycling inhalers would have much impact on the environment.	People who said that reducing their carbon footprint was “Very important” to them were nearly three times as likely to return them (See Table S1 in Supplementary Material 5 for full regression results). A behaviourally informed message, which directly connected inhaler disposal to carbon footprint, was significantly better at encouraging people to dispose of their inhalers correctly (See Table S3 in Supplementary Material 5 for full regression results). About 83% said they would probably / definitely return their inhalers in future on learning it’s the method that is better for the environment.	Recycling aligns with their personal goals of sustainability, and they had strong intentions to use a scheme (if it is simple and exists locally), That they view recycling having a positive impact
*Automatic Motivation*	Habit formation	56% of people said they dispose of their inhalers (generally inappropriately) simply out of habit.^*^ People also don’t trust themselves to remember to return their inhalers. Of the 11% who said they would likely not dispose of their inhaler correctly, 22% of this group said they would likely forget in future.	The length of time between picking up a “new” inhaler and finishing current inhaler would make it difficult to remember to recycle inhalers.	30% already attempt to recycle (incorrectly) by disposing in recycling bins Free text responses suggests that some people separate the plastics of inhalers to put what they perceive as recyclable components into recycling bin	Patients were already in the habit of going to the pharmacy to pick up prescriptions was seen as positive reinforcement to recycle Some people thought providing an incentive may help people develop the habit of recycling their inhalers.

^*^Whilst this could also be related to a lack of knowledge (psychological capability) this was coded as automatic motivation since changing a habitual way of doing something could me more challenging.

### Capability barriers

Both studies identified *psychological and physical capability* as key barriers to appropriate inhaler disposal. A key issue was low awareness or lack of knowledge about recycling inhalers, that disposal method affected resultant emissions, and insufficient guidance on how to do so. In Study 2, further *psychological capability* barriers included lack of attention to pharmacy return schemes, forgetting to recycle, and difficulty forming new recycling habits. *Physical capability* barriers were also reported. Participants in both studies cited the extra effort required to travel to a pharmacy as a deterrent, with some in Study 1 unable to make the trip independently. In Study 2, reliance on public transport was highlighted as an additional challenge. Participants also reported lacking the skills to correctly identify which parts of the inhaler could be recycled at home.

### Capability facilitators

Study 1 found that *psychological capability* significantly influenced inhaler disposal: participants who knew how to dispose of inhalers were over 16 times more likely to return them to the pharmacy, and those aware of the environmental impact were over twice as likely. Although Study 2 participants did not report any *psychological capability* facilitators, those who reported *physical capability* facilitators may have possessed an element of implied knowledge that inhalers can be recycled. For *physical capability*, Study 2 interviewees suggested that having the physical skills required to recycle (e.g. dismantle the canister) the inhaler and being able to travel to the pharmacy without extra effort would support appropriate disposal. No *physical capability facilitators* were identified in Study 1 survey data, highlighting contrasting facilitator reporting across studies.

Table 5 presents the findings relating to factors affecting participants’ opportunity of disposing of inhalers appropriately.

### Opportunity barriers

*Physical opportunity* was a notable barrier in both studies. Study 1 participants cited issues such as pharmacies being too far away, having limited opening hours, or not offering disposal services. In Study 2, those using small, independent pharmacies felt their local providers lacked the time, space, or resources to support inhaler recycling, with limited capacity emerging as a key theme. *Social opportunity* barriers were also evident. Across both studies, an important issue was the absence of advice from healthcare professionals on proper inhaler disposal. Over 80% of survey respondents in Study 1 reported not receiving or recalling any guidance, and participants in Study 2 consistently stated that no healthcare professionals (including pharmacy staff) had informed them about available recycling options.

### Opportunity facilitators

Study 1 identified potential *physical opportunity* facilitators, with older participants (who may have more frequent pharmacy visits), showing a higher likelihood of returning inhalers. Survey responses also suggested that convenient disposal points within the pharmacy setting, similar to those for batteries or vapes, would encourage recycling. Similar *physical opportunity* facilitators were reported in Study 2 interviews for those who regularly visited the pharmacy to pick up their inhalers. Regarding *social opportunity*, Study 1 highlighted that public messaging could shift social norms around inhaler disposal. In Study 2, pharmacists were viewed as trusted professionals who could play a key role in promoting inhaler recycling, emphasizing their influence in encouraging proper disposal behaviours.

Table 6 presents the findings relating to factors affecting participants’ motivation for disposing of inhalers appropriately.

### Motivation barriers

*Reflective motivation* was reported as a key barrier in both studies. In Study 1, many significantly underestimated the carbon footprint of MDIs and overestimated that of DPIs. It was reported in Study 2 that individual recycling efforts would have minimal environmental impact. Additionally, the lack of pharmacist promotion of inhaler recycling was identified as a barrier. Regarding *automatic motivation*, 56% of Study 1 participants disposed of inhalers out of habit, typically inappropriately, and many expressed doubts about remembering to return inhalers. Data from Study 2 highlighted that the long interval between receiving a new inhaler and finishing the current one made it difficult to remember proper disposal, further hindering recycling behaviour.

### Motivation facilitators

*Reflective motivation* facilitators in Study 1 revealed that participants who considered reducing their carbon footprint as “very important” were nearly three times more likely to return inhalers to pharmacies. When informed that proper inhaler disposal benefits the environment, 83% said they would “probably” or “definitely” recycle in the future. Behaviourally informed messages that linked inhaler disposal directly to carbon emissions were significantly more effective than standard messaging. In Study 2, *reflective motivation* facilitators included a belief in personal ability to recycle. However, strong intentions to engage in schemes were also dependent on it being “simple” and local (i.e. *physical opportunity*). The view that pharmacists should actively encourage recycling as part of their professional role was also evident across interviews. Positive attitudes were expressed towards inhaler recycling once informed, aligning it with personal sustainability goals. Regarding *automatic motivation*, 30% of Study 1 participants already attempted to recycle inhalers, albeit often incorrectly, such as placing parts in recycling bins at home. In Study 2, habitual pharmacy visits supported recycling behaviour, and some interviewees felt incentives might encourage habit formation, though these were not essential.

## Discussion

This paper combined datasets from two studies utilising contrasting methodologies to explore inhaler recycling awareness and behaviours through a psychological lens, drawing on the application of behavioural models. This novel approach and the merging of findings from two nations with different health systems highlighted many commonalities. The synthesis of findings highlights that patients were unaware of the need to return inhalers to pharmacies for disposal and often discarded them in domestic waste. Inhaler users viewed community pharmacies as convenient locations for recycling schemes due to their accessibility and proximity. However, they were unlikely to engage with initiatives that require significant additional effort such as traveling out of their way to return inhalers.

Disposal behaviours within the datasets aligned with existing literature indicating that most inhalers in the UK are not recycled^4,13,17,18,42^. In line with the findings of Murphy et al^7^ regarding awareness of the carbon footprint of improper inhaler disposal, Study 1 participants who understood the environmental impact of inhaler disposal were more than twice as likely to indicate a future intention to return their inhalers to a pharmacy. Overall, across both studies, *capability, opportunity, and motivation* barriers hindered effective inhaler disposal, suggesting a need for clearer guidance on what and how to recycle, improved accessibility and signposting, and interventions that highlight the benefits of recycling and support habit formation.

Despite strong intentions to engage with inhaler recycling in the future, inhaler users in both studies perceived that it would be challenging to form new disposal habits. This suggests that even though inhaler users exhibit a readiness to adopt new sustainable practices there maybe challenges in engaging long-term behaviour change. The formation of new recycling habits can be hindered by established routines which are resistant to change^43^; in this case most inhaler users were disposing of their inhalers within household bins or breaking the inhalers into component parts, so that the plastic could be picked up by local curb side recycling collections. To achieve sustained behaviour change it is important that the common barriers to recycling are addressed. Our findings underline the need for all healthcare professionals, involved in the prescribing and dispensing of inhalers to build the importance of recycling into their conversations. Promotional messages that frame inhaler recycling as a valued, simple and accessible behaviour could help encourage inhaler users to adopt new recycling habits. Further to this, Study 1 found that a behaviourally informed message, using tangible information to educate participants on the importance of proper inhaler disposal, emphasising positive environmental gains for society and using positive framing to increase self-efficacy and prevent feelings of guilt^44,45^ was more likely to prompt inhaler users to consider inhaler recycling compared to current NHS communications. A longitudinal study^46^ trialled the use of behaviourally informed information leaflets to promote recycling in Sweden, with findings highlighting that such messaging increased pro-environmental behaviour and rates of food recycling.

### Limitations and strengths

There are some limitations that may affect the generalisability of these findings and therefore should be interpreted within the context of this paper. Convenience sampling was used in both studies which may have excluded individuals with lower health engagement or digital access. This may have also led to over-representation of people who were environmentally aware or held positive values related to sustainability. Disposal behaviours and prior guidance were self‑reported and therefore subject to recall and social‑desirability bias^47^. Furthermore, this sampling approach may not have appropriately targeted groups which are underrepresented in research. Issues relating to geographic differences and devolved healthcare (e.g. free prescriptions in Wales) and distinct sampling strategies may also limit direct comparison. Neither study collected data on frequency of pharmacy visits, or polypharmacy which may have explained why older participants in Study 1 were more likely to recycle than younger inhaler users^48^.

Study 1 was conducted online, relying on NHS volunteer panels, and may have biased the sample toward those with higher health literacy and digital connectivity^49^. Both studies achieved a good response, however, low awareness among patients about the need to return inhalers to pharmacies for disposal could have impacted recruitment (e.g. low perceived relevance or reduced motivation to engage). We acknowledge that neither studies adhered to current reporting standards to distinguish between sex and gender in health research^50^. Both studies explored hypothetical behaviours, such as future willingness to dispose of inhalers properly, which may not reflect actual actions due to intention–action gaps^51^ and possible social desirability bias^47^. Additionally, Study 1 compared two different message formats, but the design does not isolate which specific features (e.g., colour, wording, layout) influenced the reported intentions, limiting interpretation of the messaging effects.

A strength of both studies lies the use of psychological frameworks to underpin the work. The systematic exploration of the barriers and facilitators by applying a behavioural diagnosis, identified which COM-B component(s) to address in the design of an intervention to support this population of inhaler users, however, the coding process can be open to interpretation. As illustrated by Pressau et al^52^., experienced coders report interpretive disagreements and the need to make explicit assumptions, underscoring the subjective element of data mapping despite clear definitions. While framework analysis indicated that all barriers and facilitators reported could be mapped to COM-B (with no additional themes identified), Nguyen et al^53^, advocate an integrated model by adding socio-ecological problems to the COM-B model which might have uncovered wider systemic issues relating to inhaler recycling in pharmacies. To mitigate this, free-text comments captured in Study 1 allowed further explanatory data to be elucidated, while the initial inductive analysis conducted in Study 2 provided further qualitative insights into the barriers and facilitators of inhaler recycling behaviours.

### Recommendations and further research

To ensure increased uptake, future schemes must be designed in a way that ensures accessibility (e.g. placing recycling bins in convenient locations within the local community). However, public awareness of inhaler recycling remains low. Most existing schemes have been limited to a single setting, typically the pharmacy. A whole-systems approach involving all patient-facing healthcare professionals and system-level changes within the broader healthcare environment is likely to be more effective in promoting lasting changes in behaviour. The BCW was used to make recommendations for intervention functions that would support future behaviour change. Behavioural insights from both studies were mapped to COM-B and were linked to their respective intervention functions on the BCW. This identified five priority areas which could support the success of future inhaler recycling schemes within community pharmacies.

1. To establish national-level educational campaigns which highlight the environmental benefits of proper inhaler disposal (Education).
2. To improve signposting to inhaler recycling schemes in the community, for example, posters in asthma clinics or adding recycling prompts to medication labelling, can help raise awareness and remind inhaler users to engage (Environmental Restructuring).
3. To support sustained engagement, inhaler users should receive feedback on the environmental impact of recycling schemes (Persuasion).
4. To adopt behaviourally informed messaging to encourage inhaler users to appropriately dispose of inhalers (Persuasion).
5. To acknowledge (or praise) those who engage in the recycling scheme (Incentivisation).

Future research should focus on investigating the impact of the above strategies on inhaler recycling uptake, harnessing a mixed-methods approach to identify effective interventions and reasons for their success or otherwise. While this study focused specifically on MDI recycling since it is the most environmentally harmful medicinal product, future research should also consider whether targeting specific environmental awareness around inhalers is the most effective approach, or whether promoting general medicine return habits to pharmacies may be more practical and sustainable. A broader ‘return all medicines’ message may be easier for patients to remember and maintain consistent behaviour, avoiding the need for product-specific education.

## Conclusion

To maximise the impact of inhaler recycling schemes, there is a need to understand what patients currently do with their inhalers once the inhaler cartridge is empty or the inhaler is no longer needed, and to explore their attitudes and beliefs about inhaler disposal. This study highlights significant gaps in awareness and behaviour regarding proper inhaler disposal across two different healthcare contexts. Despite recognising community pharmacies as convenient disposal points, many inhaler users lack knowledge about appropriate disposal methods and often discard inhalers in household waste. While environmental concerns can motivate recycling intentions, forming new, sustained habits remains difficult. The findings underscore the need for multifaceted interventions that address psychological, physical, and social barriers. By leveraging behaviour change frameworks like COM-B and the Behaviour Change Wheel, targeted strategies—such as national education campaigns, improved signposting, feedback mechanisms, persuasive messaging, and incentivization—can enhance engagement with recycling schemes. Importantly, fostering a whole-system approach involving all healthcare professionals can support sustained behaviour change and broaden reach. Future research should focus on implementing and evaluating these interventions to identify effective methods for increasing inhaler recycling uptake. Ultimately, enhancing inhaler disposal practices can contribute to reducing the environmental impact of inhaler use and promote more sustainable healthcare behaviours.

## Supplementary information

Supplementary Material 1

Supplementary Material 2 Survey and Message Design

Supplementary Material 3 Int Schedule

Supplementary Material 4_COREQ_

Supplementary Material 5 (Regression tables)

## Data Availability

The data sets generated and/or analysed during the current study are available from the corresponding author on reasonable request.
