# Supplementary material for: Patient attitudes, beliefs, and practices in environmentally friendly inhaler disposal: a mixed-methods evaluation with behaviourally informed recommendations
Source: NPJ Prim Care Respir Med. 2026 May 22;36:56. doi: 10.1038/s41533-026-00521-6 (PMC13470037; doi:10.1038/s41533-026-00521-6)
Supplement: Supplementary file 2 [file 41533_2026_521_MOESM1_ESM.docx]

**Supplementary Material 1 – Study Inhaler Disposal Survey**

**Inhaler Disposal** **Survey Flow**

**BlockRandomizer: 1 - Evenly Present Elements**

**EmbeddedData**

**order = 1**

**EmbeddedData**

**order = 2**

**Block: Consent and Screening (3 Questions)**

**Branch: New Branch**

**If**

**If NHS England is inviting you to take part in a survey of inhaler users in England. This will help... I do not consent to taking part in this survey Is Selected**

**Or This survey is intended for adults who live in England I don't live in England and/or I am under 18 Is Selected**

**Or Do you currently use an inhaler, or are you the guardian of someone who does? No, neither of these apply to me Is Selected**

**EndSurvey: Advanced**

**Standard: Current Practice and Awareness of Best Practice (6 Questions)**

**BlockRandomizer: 1 - Evenly Present Elements**

**Standard: Message B (2 Questions)**

**Standard: Message A (2 Questions)**

**Standard: carbonFootprintIntro (1 Question)**

**BlockRandomizer: 1 - Evenly Present Elements**

**Block: Awareness of Environmental Impact - MDI first (2 Questions)**

**Block: Awareness of Environmental Impact - DPI first (2 Questions)**

**Standard: Answer (1 Question)**

**BlockRandomizer: 1 - Evenly Present Elements**

**Block: recyclingQuestionA (3 Questions)**

**Block: recyclingQuestionB (3 Questions)**

**Standard: Inhaler General Background Questions (7 Questions)**

**Block: Background questions (6 Questions)**

**EndSurvey: Default**

Informed Consent NHS England is inviting you to take part in a survey of inhaler users in England. This will help NHS England to improve how we communicate to patients. No personally identifying data will be collected and all responses will be anonymous. Your data will be stored securely and analysed by NHS England staff only. It will be deleted 3 years after the end of the project. The reports we develop by analysing your data may be shared with other NHS England project teams and adapted for publication in academic journals. If you have any questions about this research, please contact [england.bsci@nhs.net](mailto:england.bsci@nhs.net). You can change your mind and decide not to take part at any time before your responses are submitted. The survey should take 5-10 minutes to complete. Anyone who lives in England, is 18 or over and uses an inhaler or is the guardian of someone who uses an inhaler can take part. The survey will involve questions about your current inhaler usage and disposal methods, as well as some on your lifestyle and health.

**Consent form**

1. I have read the information above, and I understand what the survey will involve.

2. I understand that taking part is voluntary and I can withdraw at any time without giving a reason. Please click on one of the boxes below to show your consent, and then the arrow to continue.

- I have read the above information and consent to taking part in this survey (1)
- I do not consent to taking part in this survey (0)

This survey is intended for adults who live in England

- I live in England and am 18 or over (1)
- I don't live in England and/or I am under 18 (0)

Do you currently use an inhaler, or are you the guardian of someone who does?

- Yes, I use an inhaler (1)
- Yes, I am a guardian of someone who uses one (2)
- No, neither of these apply to me (3)

**We are now going to ask you some questions about how you dispose of your inhaler.** How do you dispose of your inhaler?

- General waste/ Bin (1)
- Recycling bin (2)
- Return to the pharmacy (3)
- Take to the hospital (4)
- Don't know/ Can't remember (6)
- Other (5) __________________________________________________

*Display this question:*

*If We are now going to ask you some questions about how you dispose of your inhaler. How do you disp... = General waste/ Bin*

You said that you dispose of your inhaler in the general waste. Why do you do this? Please select all the reasons that apply:

▢ It's how I have always disposed of them (1)
▢ It's the easiest way to dispose of them (2)
▢ It's how I'm supposed to dispose of them (3)
▢ That's what I've been told to do by my friends/family (4)
▢ That's what I've been told to do by a healthcare professional (5)
▢ Other (6) __________________________________________________

*Display this question:*

*If We are now going to ask you some questions about how you dispose of your inhaler. How do you disp... = Recycling bin*

You said that you dispose of your inhaler in the recycling bin. Why do you do this? Please select all the reasons that apply:

▢ It's how I have always disposed of them (1)
▢ It's the easiest way to dispose of them (2)
▢ I think it's how I'm supposed to dispose of them (3)
▢ That's what I've been told to do by my friends/family (4)
▢ That's what I've been told to do by a healthcare professional (5)
▢ I don't know (7)
▢ Other (6) __________________________________________________

*Display this question:*

*If We are now going to ask you some questions about how you dispose of your inhaler. How do you disp... = Return to the pharmacy*

You said that you dispose of your inhaler by returning it to the pharmacy. Why do you do this? Please select all the reasons that apply:

▢ It's how I have always disposed of them (1)
▢ It's the easiest way to dispose of them (2)
▢ I think it's how I'm supposed to dispose of them (3)
▢ That's what I've been told to do by my friends/family (4)
▢ That's what I've been told to do by a healthcare professional (5)
▢ I don't know (7)
▢ Other (6) _________________________________________________*isplay this question:*

*If We are now going to ask you some questions about how you dispose of your inhaler. How do you disp... = Take to the hospital*

You said that you dispose of your inhaler by bringing it to the hospital. Why do you do this? Please select all the reasons that apply:

▢ It's how I have always disposed of them (1)
▢ It's the easiest way to dispose of them (2)
▢ I think it's how I'm supposed to dispose of them (3)
▢ That's what I've been told to do by my friends/family (4)
▢ That's what I've been told to do by a healthcare professional (5)
▢ I don't know (7)
▢ Other (6) __________________________________________________

How do you think the **NHS recommends** that you dispose of your inhaler?

- General waste/ bin (1)
- Recycling bin (2)
- Return to the pharmacy (3)
- Take to the hospital (4)
- Don't know (6)
- Other (5) __________________________________________________

**Please imagine that you see this message on the NHS website:** After viewing this message, how likely would you be to return your inhalers to your pharmacy in the future?

- Definitely Not (1)
- Probably Not (2)
- Somewhat unlikely (3)
- Unsure (4)
- Somewhat likely (5)
- Probably (6)
- Definitely (7)

*Display this question:*

*If Please imagine that you see this message on the NHS website: After viewing this message, how like... = Definitely Not*

*Or Please imagine that you see this message on the NHS website: After viewing this message, how like... = Probably Not*

*Or Please imagine that you see this message on the NHS website: After viewing this message, how like... = Somewhat unlikely*

*Or Please imagine that you see this message on the NHS website: After viewing this message, how like... = Unsure*

You indicated that you were not likely to return your inhaler to the pharmacy. Why wouldn't you return your inhaler to the pharmacy? Select all that apply

▢ It's too much extra effort (1)
▢ I don't see the point (2)
▢ I'm in the habit of disposing of it another way (3)
▢ I don't believe it will make a difference to the environment (4)
▢ I don't believe that our actions contribute to climate change (5)
▢ It goes against the advice I've been given in the past (6)
▢ Someone else disposes of my inhalers for me (7)
▢ I might forget to do it (8)
▢ Other (9) __________________________________________________

**Please imagine that you see this message on the NHS website:** After viewing this message, how likely would you be to return your inhalers to your pharmacy in the future?

- Definitely Not (1)
- Probably Not (2)
- Somewhat unlikely (3)
- Unsure (4)
- Somewhat likely (5)
- Probably (6)
- Definitely (7)

*Display this question:*

*If Please imagine that you see this message on the NHS website: After viewing this message, how like... = Definitely Not*

*Or Please imagine that you see this message on the NHS website: After viewing this message, how like... = Probably Not*

*Or Please imagine that you see this message on the NHS website: After viewing this message, how like... = Somewhat unlikely*

*Or Please imagine that you see this message on the NHS website: After viewing this message, how like... = Unsure*

You indicated that you were not likely to return your inhaler to the pharmacy. Why wouldn't you return your inhaler to the pharmacy? Select all that apply

▢ It's too much extra effort (1)
▢ I don’t see the point (2)
▢ I'm in the habit of disposing of it another way (3)
▢ I don’t believe it will make a difference to the environment (4)
▢ I don’t believe that our actions contribute to climate change (5)
▢ It goes against the advice I’ve been given in the past (6)
▢ Someone else disposes of my inhalers for me (7)
▢ I might forget to do it (8)
▢ Other (9) __________________________________________________

**We are now going to ask you some questions about the carbon footprint of some commonly used inhalers.**

How many miles do you need to drive in a car to have the same carbon footprint of the inhaler shown above? (The inhaler shown is a standard pressurised metered dose inhaler (MDI) which will last around 100 uses. The car in this example is an average petrol car.)

How many miles do you need to drive in a car to have the same carbon footprint of the inhaler shown above? (The inhaler shown is a standard dry powder inhaler (DPI) which will last around 60 uses. The car in this example is an average petrol car.)

How many miles do you need to drive in a car to have the same carbon footprint of the inhaler shown above? (The inhaler shown is a standard dry powder inhaler (DPI) which will last around 60 uses. The car in this example is an average petrol car.)

How many miles do you need to drive in a car to have the same carbon footprint of the inhaler shown above? (The inhaler shown is a standard pressurised metered dose inhaler (MDI) which will last around 100 uses. The car in this example is an average petrol car.)

**Answer** The metered dose inhaler (Ventolin Evohaler) has a carbon footprint equal to 107 miles in an average petrol car, the dry powder inhaler (Ventolin Accuhaler) has a carbon footprint equal to 2.1 miles. The carbon footprint of the metered dose inhaler is over **48 times greater.**

Inhalers typically contain potent greenhouse gases as propellants. When disposed of incorrectly, such as being thrown in the regular bin, these gases are released into the atmosphere and contribute to global warming.

If you were told that, by returning your used inhalers to the pharmacy: **They would undergo thermal treatment, such as incineration, to destroy the greenhouse gases and prevent inhaler plastics from ending up in landfills** How likely would you be to bring your used inhaler back to the pharmacy?

- Definitely Not (1)
- Probably Not (2)
- Somewhat Unlikely (3)
- Unsure (4)
- Somewhat Likely (5)
- Probably (6)
- Definitely (7)

If you were told that, by returning your used inhalers to the pharmacy: **The canisters and plastic components would be recycled and reused, and any remaining gases would be extracted and reused in items like fridges and air conditioning units** How likely would you be to bring your used inhaler back to the pharmacy?

- Definitely Not (1)
- Probably Not (2)
- Somewhat Unlikely (3)
- Unsure (4)
- Somewhat Likely (5)
- Probably (6)
- Definitely (7)

Inhalers typically contain potent greenhouse gases as propellants. When disposed of incorrectly, such as being thrown in the regular bin, these gases are released into the atmosphere and contribute to global warming.

If you were told that, by returning your used inhalers to the pharmacy: **The canisters and plastic components would be recycled and reused, and any remaining gases would be extracted and reused in items like fridges and air conditioning units** How likely would you be to bring your used inhaler back to the pharmacy?

- Definitely Not (1)
- Probably Not (2)
- Somewhat Unlikely (3)
- Unsure (4)
- Somewhat Likely (5)
- Probably (6)
- Definitely (7)

If you were told that, by returning your used inhalers to the pharmacy: **They would undergo thermal treatment, such as incineration, to destroy the greenhouse gases and prevent inhaler plastics from ending up in landfills** How likely would you be to bring your used inhaler back to the pharmacy?

- Definitely Not (1)
- Probably Not (2)
- Somewhat Unlikely (3)
- Unsure (4)
- Somewhat Likely (5)
- Probably (6)
- Definitely (7)

What type of inhaler do you use? Tick all that apply

▢ Metered Dose Inhaler (1)
▢ Dry Powder Inhaler (2)
▢ Soft Mist Inhaler (3)
▢ Other (4) __________________________________________________
▢ Unsure (5)

Have you received guidance on how best to dispose of your inhalers?

- Yes – I dispose of my inhaler as instructed every time (2)
- Yes – and I sometimes dispose of my inhaler as instructed (3)
- Yes – but I do not dispose of my inhaler as instructed (4)
- Yes – but I have forgotten how I was told to dispose of my inhaler (5)
- No – I have never received guidance on how to dispose of my inhaler (1)
- I don't know (6)

*Display this question:*

*If Have you received guidance on how best to dispose of your inhalers? = Yes – I dispose of my inhaler as instructed every time*

*Or Have you received guidance on how best to dispose of your inhalers? = Yes – and I sometimes dispose of my inhaler as instructed*

*Or Have you received guidance on how best to dispose of your inhalers? = Yes – but I do not dispose of my inhaler as instructed*

*Or Have you received guidance on how best to dispose of your inhalers? = Yes – but I have forgotten how I was told to dispose of my inhaler*

Who provided you with the guidance on how to dispose of your inhaler?

- The pharmacist (1)
- My GP (2)
- A nurse practitioner (3)
- A respiratory therapist (4)
- Online resources (5)
- I don't know (7)
- Other (6) __________________________________________________

Before taking part in this survey, were you aware that the carbon footprint of your inhaler changes based on your disposal method?

- Yes (1)
- No (0)

How important is it for you to reduce the carbon footprint of your inhaler usage?

- Not important (1)
- Slightly important (2)
- Moderately Important (3)
- Important (4)
- Very Important (5)

What support would you need to change to a low carbon footprint disposal method? Tick all that apply

▢ More information from a clinical professional when given the inhaler (1)
▢ More convenient point of disposal (e.g. knowledge that all pharmacies and supermarkets accepted inhales) (2)
▢ Being able to return inhalers in the post (3)
▢ I would not change my method of disposal (5)
▢ Other (4) __________________________________________________

If all of the information was made available to you, would you consider switching to a different disposal method for environmental reasons?

- Yes (2)
- No (1)
- I don't know (3)

Finally, we'd like to ask a few questions about you.

How old are you?

Which of the following best describes your gender?

- Male (1)
- Female (2)
- Non-binary (3)
- Prefer to self-describe (4) __________________________________________________
- Prefer not to say (5)

What is your ethnic group?

- White (1)
- Asian or Asian British (2)
- Black, Black British, Caribbean or African (3)
- Mixed or Multiple ethnic groups (4)
- Other (5) __________________________________________________
- Prefer not to say (6)

What is the highest level of education you have completed?

- Secondary School (2)
- Vocational or similar (3)
- Undergraduate degree (4)
- Postgraduate or professional degree (5)
- None (1)
- Prefer not to say (6)

What health condition do you use your inhaler for? Tick all that apply

▢ Asthma (1)
▢ Chronic obstructive pulmonary disease (COPD) (2)
▢ Other (3) __________________________________________________
▢ Prefer not to say (4)

**Supplementary Material 2 – Study 2 Inhaler Users (Guardians) Interview Schedule**

**Introductory Questions**

1. Which community pharmacy do you mainly use?
2. Are you aware of whether this community pharmacy has an inhaler-recycling scheme? If you were to create an inhaler-recycling scheme, what would it look like?
3. Does your community pharmacy have a recycling scheme for any other medication?
4. Are you aware of the environmental impact that the gases within inhalers have on the environment? Could you tell me what you know about it?

**Table 1: Interview Questions based on the COM-B Model and Theoretical Domains Framework.**

| **COM-B MODEL** | **THEORETICAL DOMAINS FRAMEWORK** | **QUESTION** |
| --- | --- | --- |
| **PSYCHOLOGICAL**  **CAPABILITY** | Knowledge | What methods and/or processes of  inhaler recycling do you know of, if any? |
|  | Memory, attention and decision process | How easy or difficult is it to remember to bring back your empty/unwanted inhalers back to the pharmacy? |
|  | Behaviour regulation | Do you ever receive advice about the disposal of inhalers by community pharmacy staff?  In terms of recycling, do you ever receive feedback on your performance of inhaler disposal from community pharmacy staff? |
| **PHYSICAL CAPABILITY** | Skills | How do you dispose of your inhalers? |
| **SOCIAL OPPORTUNITY** | Social influences | Does community pharmacy staff guide you on how you should recycle and correctly dispose of  your inhalers? |
| **PHYSICAL OPPORTUNITY** | Environmental context and resources | To what extent does your pharmacy environment have sufficient resources to:   1. Inform you about what you should do with your used/unwanted inhalers.   And   2. Recycle your inhalers. |
| **REFLECTIVE MOTIVATION** | Social/professional role and identity | To what extent do you consider performing the steps in the correct disposal of inhalers a part of your role? |
|  | Belief about capability | Are there any steps of returning your inhaler back to the pharmacy that you are more or less confident about performing (and why)?  What step(s) would you be most/least confident about performing (and why)? |
|  | Optimism | How optimistic or pessimistic are you that you would return your inhalers back to the pharmacy for correct disposal and recycling in the  future? |
|  | Intention | To what extent do you intend to correctly recycle your inhalers by returning them back to your pharmacy? |
|  | Goals | Do you have any specific goals revolving around inhaler recycling? Do you have any specific goals for improving sustainability through  correct inhaler disposal? |
|  | Belief about consequences | To what extent do you believe that returning your inhaler back to the pharmacy for recycling could  improve sustainability both now and in the future? |
| **AUTOMATIC MOTIVATION** | Reinforcement | Are you aware of any rewards given to service users for returning their inhalers to pharmacies to be recycled?  Would you be more likely to return your inhalers back to the pharmacy if there was a reward scheme in  place? |
|  | Emotion | Why don’t you return your inhalers back to the pharmacy to be recycled?  To what extent do you feel that your emotional state towards sustainability affects your performance of inhaler disposal? |
