# Supplementary material for: Patient attitudes, beliefs, and practices in environmentally friendly inhaler disposal: a mixed-methods evaluation with behaviourally informed recommendations
Source: NPJ Prim Care Respir Med. 2026 May 22;36:56. doi: 10.1038/s41533-026-00521-6 (PMC13470037; doi:10.1038/s41533-026-00521-6)
Supplement: Supplementary file 3 [file 41533_2026_521_MOESM2_ESM.docx]

**Supplementary Material 2 – Study 1 questionnaire and stimuli design**

**Questionnaire development and Validation**

A multi‑stage quantitative questionnaire was developed to capture current inhaler disposal behaviours, their understanding of recommended disposal practices, perceived barriers and facilitators to returning inhalers, and their responses to different communication messages on inhaler disposal. Questionnaire items were generated by adapting of established items identified from previous surveys of inhaler use and disposal, and by designing new items to address gaps in the literature.

To ensure content and face validity, the draft questionnaire was piloted with a convenience sample of ten adult inhaler users. Participants completed the full instrument and provided structured feedback on clarity, comprehension, response burden, and perceived relevance. Minor refinements were made to improve wording clarity and the logic of conditional questions. This process ensured that the questionnaire was understandable to the intended population and adequately captured key behavioural constructs prior to large‑scale administration.

**Theoretical basis: COM‑B and Behaviour Change Wheel**

The questionnaire was structured around the COM‑B model (Capability, Opportunity, Motivation → Behaviour) (Michie et al., 2011). Items were designed to capture factors informing inhaler disposal behaviours:

- **Psychological Capability**: knowledge of recommended disposal, awareness of environmental impact, carbon footprint‑ literacy, and prior guidance received.
- **Physical Capability**: perceived effort required to return inhalers and practical difficulties associated with pharmacy visits.
- **Physical Opportunity**: environmental constraints, availability of disposal options, and preferences for more convenient return mechanisms.
- **Social Opportunity**: influence of healthcare professionals and social norms.
- **Reflective Motivation**: beliefs about environmental consequences, importance of reducing inhaler emissions, and intentions formed after message exposure.
- **Automatic Motivation**: habitual routines, forgetfulness, and entrenched patterns of disposal.

These constructs directly informed both item design (see Table 2) and the selection of message content for the experiment evaluating communication strategies.

***Table 2. Summary of questionnaire items, evidence base, novelty, and COM‑B domain***

| **Survey Item / Construct** | **Basis in prior literature at time of questionnaire design** | **COM‑B Domain** |
| --- | --- | --- |
| Disposal method of inhalers (MCQ) | Yes – similar items exist (Murphy et al., 2023; Quantz et al., 2023; Herrera et al., 2024) | *Automatic Motivation*, *Reflective Motivation*, *Physical Opportunity* |
| Reasons for chosen disposal method | No equivalent identified | *Automatic Motivation*, *Reflective Motivation*, *Physical Capability* |
| Perceived NHS disposal guidance | No equivalent identified | *Psychological Capability* |
| Likelihood of returning inhalers in future after presentation of message | No equivalent identified | *Reflective Motivation* |
| Barriers to returning inhalers | No equivalent identified | *Physical Capability*, *Automatic Motivation*, *Reflective Motivation* |
| Awareness of inhaler environmental impact – Carbon ‑footprint equivalence (miles vs. inhaler) | Similar items exist assessing awareness (Florman et al., 2022; Quantz et al., 2023; Herrera et al., 2024), but none using this method | *Psychological Capability* |
| Likelihood to return inhalers for thermal treatment | No analogue | *Reflective Motivation* |
| Likelihood to return inhalers for recycling | No analogue | *Reflective Motivation* |
| Types of inhalers used | Yes – similar items exist (Murphy et al., 2023; Quantz et al., 2023) | *Physical Opportunity* |
| Guidance received on disposal | Similar items exist on proper inhaler use, but not disposal (Quantz et al., 2023) | *Social Opportunity*, *Psychological Capability* |
| Source of disposal guidance | Similar items exist on proper inhaler use, but not disposal (Quantz et al., 2023) | *Social Opportunity* |
| Awareness that disposal method affects emissions | Similar items exist, but not related to disposal method (Florman et al., 2022; Herrera et al., 2024) | *Psychological Capability* |
| Importance of reducing inhaler carbon footprint | Yes – similar items exist (Florman et al., 2022; Quantz et al., 2023) | *Reflective Motivation* |
| Support needed to switch disposal method | Similar items exist on switching inhaler type, but not related to disposal method (Quantz et al., 2023) | *Physical Opportunity*, *Social Opportunity* |

**Messaging Stimuli Design**

**Purpose of the messaging component**

The messaging experiment aimed to test whether communication content aligned with behavioural science principles could increase people’s stated likelihood of returning inhalers to community pharmacies. Participants were randomly assigned to receive either a baseline NHS communication (Message A) or a behaviourally informed message (Message B), after which they reported their likelihood of returning inhalers.

**Message A: Standard NHS Communication**

Message A was designed to closely mirror NHS advice on inhaler disposal. The wording was taken directly from guidance provided on the NHS Asthma Treatment page at the time of the study design. This instructed patients to return used or expired inhalers to a pharmacy for appropriate disposal, and it reflected the typical NHS blue colour palette for health communications.

Being based on this official guidance, Message A did not include explicit explanation of the environmental rationale behind the guidance.

**Message B: Behaviourally Informed Communication**

Message B was developed as an enhanced communication stimulus using principles drawn from the Behaviour Change Wheel, COM-B, and the environmental‑ behaviour literature. It used Greener NHS green to visually distinguish environmentally focused messaging and incorporated multiple evidence-based behaviour change strategies:

- **Tangible, relatable information and education**: Provided a clear and specific explanation of *why* returning inhalers matters, linking disposal to carbon footprint reduction in a tangible and relatable way (Rau et al., 2022)
- **Actionable, practical steps to increase self‑efficacy**: Presented clear, simple instructions to reduce perceived effort and increase perceived behavioural control (Schutte & Bhullar, 2017).
- **Positive framing to prevent cognitive dissonance / guilt**: Used positive, gain‑framed messages reduce defensiveness and avoid triggering maladaptive guilt responses, making pro‑environmental messaging more likely to be accepted (Grazzini et al., 2018).
- **General positive environmental gains for society**: Emphasised collective benefit (“our environment”) to encourage pro-environmental social norm (Grilli & Curtis, 2021).
- **Accessibility**: Plain language to improve comprehension across literacy levels, enabling individuals to understand recommended actions and increasing the likelihood of behaviour change (Grene et al., 2017).

Message B thus represented a composite, policy relevant behaviour change intervention, designed not to isolate individual components but to evaluate the effect of an integrated, evidence‑ based communication package relative to the existing NHS standard.

**Awareness of inhaler environmental impact**

**Purpose of the estimation**

In order to assess public awareness of the environmental impact of metered dose inhalers (MDIs) and dry powder inhalers (DPIs), we asked participants to estimate the carbon footprint of a popular MDI and equivalent DPI in terms of miles driven in an average petrol car. This was to give an idea of the degree to which inhaler users overestimate or underestimate the carbon footprint of the two inhalers types.

For clarity and comparability, the CO₂e emissions from inhalers were converted into behavioural carbon equivalents, expressed as the distance a typical petrol car would travel to produce an equivalent greenhouse gas impact. This was done as people generally find it easier to contextualise the emissions in more familiar units, such as miles driven in a petrol car or full smartphone charges (Lawson et al., 2025).

**Conversion of Emissions**

Taking a popular MDI (Ventolin Evohaler) and an equivalent DPI (Ventolin Accuhaler) indicative carbon footprint g CO₂e (PrescQIPP, n.d., Attachment 1, Table Inhaler CF Full, version 2.29), we converted these to miles driven in an average petrol car (Department for Energy Security and Net Zero, 2023). This allowed us to compare estimates from participants to the actual answer.

References

Department for Energy Security and Net Zero. (2023). *Greenhouse gas reporting: Conversion factors 2023*. <https://www.gov.uk/government/publications/greenhouse-gas-reporting-conversion-factors-2023>

Florman, K., Wickham, H., Hodge, A., Umastuthan, R., Yeoh, S. L., Pang, J., ... & Patel, A. (2022). S65 global warming impact of inhalers: the patient perspective. *Thorax*, *77*(Suppl 1), A41-A41.

Grazzini, L., Rodrigo, P., Aiello, G., & Viglia, G. (2018). Loss or gain? The role of message framing in hotel guests’ recycling behaviour. *Journal of Sustainable Tourism*, *26*(11), 1944-1966.

Grene, M., Cleary, Y., & Marcus-Quinn, A. (2017). Use of plain-language guidelines to promote health literacy. *IEEE Transactions on Professional Communication*, *60*(4), 384-400.

Grilli, G., & Curtis, J. (2021). Encouraging pro-environmental behaviours: A review of methods and approaches. *Renewable and Sustainable Energy Reviews*, *135*, 110039.

Herrera, Helena, M. Charalambou, H. Dean, D. Youles, Nick Warren, and Paul Rutter. "Patients' views and behaviours in relation to asthma inhalers use and their impact on environmental sustainability: a cross-sectional survey study." *International Journal of Pharmacy Practice* 32, no. Supplement_1 (2024): i35.

Lawson, B. T., Coulentianos, M., Vrain, E., & Mitchell, V. (2025). CO2e (best) avoided? How people experience CO2e avoided on the Too Good To Go app. *Big Data & Society*, *12*(4).

Murphy, A., Carroll, W., Gotsell, M., Potter, C., Quint, J., & Malone, R. (2023). P46 How and when do patients dispose of old or unwanted inhalers?.

McGuire, N. M. (2015). Environmental education and behavioral change: An identity-based environmental education model. *International Journal of Environmental and Science Education*, *10*(5), 695-715.

PrescQIPP. (2023). *Bulletin 375: Inhaler carbon footprint*. <https://www.prescqipp.info/our-resources/bulletins/bulletin-375-inhaler-carbon-footprint/>

Quantz, D., Wong, G. Y., & Liang, K. (2023). Patient perspectives on the environmental impact of inhalers: a survey in British Columbia. *Canadian Pharmacists Journal/Revue des Pharmaciens du Canada*, *156*(6), 298-302.

Rau, H., Nicolai, S., & Stoll-Kleemann, S. (2022). A systematic review to assess the evidence-based effectiveness, content, and success factors of behavior change interventions for enhancing pro-environmental behavior in individuals. *Frontiers in Psychology*, *13*, 901927.

Schutte, N. S., & Bhullar, N. (2017). Approaching environmental sustainability: Perceptions of self-efficacy and changeability. *The Journal of Psychology*, *151*(3), 321-333.
