# Supplementary material for: Patient attitudes, beliefs, and practices in environmentally friendly inhaler disposal: a mixed-methods evaluation with behaviourally informed recommendations
Source: NPJ Prim Care Respir Med. 2026 May 22;36:56. doi: 10.1038/s41533-026-00521-6 (PMC13470037; doi:10.1038/s41533-026-00521-6)
Supplement: Supplementary file 4 [file 41533_2026_521_MOESM3_ESM.docx]

**Supplementary Material 3 – Study 2 Inhaler Users (Guardians) Interview Schedule**

**Introductory Questions**

1. Which community pharmacy do you mainly use?
2. Are you aware of whether this community pharmacy has an inhaler-recycling scheme? If you were to create an inhaler-recycling scheme, what would it look like?
3. Does your community pharmacy have a recycling scheme for any other medication?
4. Are you aware of the environmental impact that the gases within inhalers have on the environment? Could you tell me what you know about it?

**Table 1: Interview Questions based on the COM-B Model and Theoretical Domains Framework.**

| **COM-B MODEL** | **THEORETICAL DOMAINS FRAMEWORK** | **QUESTION** |
| --- | --- | --- |
| **PSYCHOLOGICAL**  **CAPABILITY** | Knowledge | What methods and/or processes of  inhaler recycling do you know of, if any? |
|  | Memory, attention and decision process | How easy or difficult is it to remember to bring back your empty/unwanted inhalers back to the pharmacy? |
|  | Behaviour regulation | Do you ever receive advice about the disposal of inhalers by community pharmacy staff?  In terms of recycling, do you ever receive feedback on your performance of inhaler disposal from community pharmacy staff? |
| **PHYSICAL CAPABILITY** | Skills | How do you dispose of your inhalers? |
| **SOCIAL OPPORTUNITY** | Social influences | Does community pharmacy staff guide you on how you should recycle and correctly dispose of  your inhalers? |
| **PHYSICAL OPPORTUNITY** | Environmental context and resources | To what extent does your pharmacy environment have sufficient resources to:   1. Inform you about what you should do with your used/unwanted inhalers.   And   2. Recycle your inhalers. |
| **REFLECTIVE MOTIVATION** | Social/professional role and identity | To what extent do you consider performing the steps in the correct disposal of inhalers a part of your role? |
|  | Belief about capability | Are there any steps of returning your inhaler back to the pharmacy that you are more or less confident about performing (and why)?  What step(s) would you be most/least confident about performing (and why)? |
|  | Optimism | How optimistic or pessimistic are you that you would return your inhalers back to the pharmacy for correct disposal and recycling in the  future? |
|  | Intention | To what extent do you intend to correctly recycle your inhalers by returning them back to your pharmacy? |
|  | Goals | Do you have any specific goals revolving around inhaler recycling? Do you have any specific goals for improving sustainability through  correct inhaler disposal? |
|  | Belief about consequences | To what extent do you believe that returning your inhaler back to the pharmacy for recycling could  improve sustainability both now and in the future? |
| **AUTOMATIC MOTIVATION** | Reinforcement | Are you aware of any rewards given to service users for returning their inhalers to pharmacies to be recycled?  Would you be more likely to return your inhalers back to the pharmacy if there was a reward scheme in  place? |
|  | Emotion | Why don’t you return your inhalers back to the pharmacy to be recycled?  To what extent do you feel that your emotional state towards sustainability affects your performance of inhaler disposal? |
