# Supplementary material for: Patient attitudes, beliefs, and practices in environmentally friendly inhaler disposal: a mixed-methods evaluation with behaviourally informed recommendations
Source: NPJ Prim Care Respir Med. 2026 May 22;36:56. doi: 10.1038/s41533-026-00521-6 (PMC13470037; doi:10.1038/s41533-026-00521-6)
Supplement: Supplementary file 5 [file 41533_2026_521_MOESM5_ESM.docx]

**Supplementary Material 5 – Study 1 Regression Tables**

#### Table S1: Binary logistic regression on current correct inhaler disposal

|  | | Odds Ratio | 95% confidence interval |
| --- | --- | --- | --- |
| Age |  | **1.02*** | [1.00, 1.04] |
|  |  |  |  |
| Gender *(Ref: Male)* |  | 1.19 | [.76, 1.88] |
|  |  |  |  |
| Ethnicity *(Ref: Caucasian)* |  |  |  |
| Asian or Asian British |  | .29 | [.07, 1.09] |
| Black British, Caribbean or African |  | 1.30 | [.29, 5.25] |
| Mixed or Multiple ethnic groups |  | .82 | [.13, 3.97] |
|  |  |  |  |
| Education *(Ref: No degree)* |  | .78 | [.51, 1.19] |
|  |  |  |  |
| Awareness of effect of disposal on carbon footprint *(Ref: Unaware)* |  | **2.21**** | [1.35, 3.62] |
|  |  |  |  |
| Importance of reducing carbon footprint *(Ref: 1-4 on 7-point importance scale)* |  | **2.80***** | [1.77, 4.50] |
|  |  |  |  |
| Knowledge of correct disposal method *(Ref: Unaware of how to dispose properly)* |  | **16.79***** | [10.89, 26.45] |
|  |  |  |  |
| Disposal training *(Ref: No training)* |  | **9.40***** | [5.96, 15.02] |

**p* < 0.05, ***p* < 0.01; ****p* < 0.001;

n = 1,105; analyses are based on complete cases, with exclusions due to participant attrition and missing data

Binary outcome variable:

0 = Not currently disposing of inhalers correctly (not returning them to the pharmacy)

1 = Currently disposing of inhalers correctly (returning them to the pharmacy)

#### Table S2: Binary logistic regression on awareness of correct inhaler disposal method

|  | | Odds Ratio | 95% confidence interval |
| --- | --- | --- | --- |
| Age |  | **.98*** | [.97, 1.00] |
|  |  |  |  |
| Gender *(Ref: Male)* |  | 1.35 | [.95, 1.95] |
|  |  |  |  |
| Ethnicity *(Ref: Caucasian)* |  |  |  |
| Asian or Asian British |  | .94 | [.35, 2.36] |
| Black British, Caribbean or African |  | .43 | [.12, 1.35] |
| Mixed or Multiple ethnic groups |  | .45 | [.10, 1.54] |
|  |  |  |  |
| Education *(Ref: No degree)* |  | 1.26 | [.90, 1.77] |
|  |  |  |  |
| Awareness of effect of disposal on carbon footprint *(Ref: Unaware)* |  | **1.93**** | [1.24, 2.98] |
|  |  |  |  |
| Importance of reducing carbon footprint *(Ref: 1-4 on 7-point importance scale)* |  | .71 | [.50, 1.01] |
|  |  |  |  |
| Current disposal method *(Ref: Incorrect current disposal)* |  | **17.77***** | [11.47, 28.12] |
|  |  |  |  |
| Disposal training *(Ref: No training)* |  | **3.39***** | [2.16, 5.30] |

**p* < 0.05, ***p* < 0.01; ****p* < 0.001;

n = 1,105; analyses are based on complete cases, with exclusions due to participant attrition and missing data

Binary outcome variable:

0 = Failed to correctly identify the NHS-recommended method of inhaler disposal

1 = Correctly identified the NHS-recommended method of inhaler disposal

#### Table S3: Binary logistic regression on likelihood to return inhaler to pharmacy in future

|  | | Odds Ratio | 95% confidence interval |
| --- | --- | --- | --- |
| Age |  | 1.01 | [.99, 1.03] |
|  |  |  |  |
| Gender *(Ref: Male)* |  | 1.29 | [.83, 2.03] |
|  |  |  |  |
| Ethnicity *(Ref: Caucasian)* |  |  |  |
| Asian or Asian British |  | 3.47 | [.62, 65.58] |
| Black British, Caribbean or African |  | 2.65 | [.48, 49.65] |
| Mixed or Multiple ethnic groups |  | .82 | [.20, 5.57] |
|  |  |  |  |
| Education *(Ref: No degree)* |  | 1.23 | [.79, 1.91] |
|  |  |  |  |
| Awareness of effect of disposal on carbon footprint *(Ref: Unaware)* |  | .63 | [.34, 1.23] |
|  |  |  |  |
| Importance of reducing carbon footprint *(Ref: 1-4 on 7-point importance scale)* |  | **6.75***** | [4.06, 11.77] |
|  |  |  |  |
| Current disposal method *(Ref: Incorrect current disposal)* |  | **28.07**** | [5.46, 516.84] |
|  |  |  |  |
| Knowledge of correct disposal method *(Ref: Unaware of how to dispose properly)* |  | .94 | [.54, 1.66] |
|  |  |  |  |
| Disposal training *(Ref: No training)* |  | 1.03 | [.47, 2.45] |
| Intervention message (Ref: Message A – control) |  | **1.61*** | [1.04, 2.50] |
|  |  |  |  |

**p* < 0.05, ***p* < 0.01; ****p* < 0.001;

n = 1,105; analyses are based on complete cases, with exclusions due to participant attrition and missing data

Binary outcome variable:

0 = Not likely to return inhalers to the pharmacy in future (1-4 on 7-point likelihood scale)

1 = Likely to return inhalers to the pharmacy in future (5-7 on 7-point likelihood scale)
